# Current State and Future Directions of Multimodality Imaging in Cardiac Sarcoidosis

**DOI:** 10.3389/fcvm.2021.785279

**Published:** 2022-01-27

**Authors:** Alison L. Wand, Jonathan Chrispin, Elie Saad, Monica Mukherjee, Allison G. Hays, Nisha A. Gilotra

**Affiliations:** ^1^Division of Cardiology, Department of Medicine, Johns Hopkins University School of Medicine, Baltimore, MD, United States; ^2^Department of Radiology, Johns Hopkins University School of Medicine, Baltimore, MD, United States

**Keywords:** cardiac sarcoidosis, sarcoid cardiomyopathy, multimodality imaging, inflammatory cardiomyopathy, echocardiography, cardiac PET, cardiac MRI (CMR)

## Abstract

Cardiac sarcoidosis (CS) is an increasingly recognized cause of heart failure and arrhythmia. Historically challenging to identify, particularly in the absence of extracardiac sarcoidosis, diagnosis of CS has improved with advancements in cardiac imaging. Recognition as well as management may require interpretation of multiple imaging modalities. Echocardiography may serve as an initial screening study for cardiac involvement in patients with systemic sarcoidosis. Cardiac magnetic resonance imaging (CMR) provides information on diagnosis as well as risk stratification, particularly for ventricular arrhythmia in the setting of late gadolinium enhancement. More recently, ^18^F-fluorodeoxyglucose position emission tomography (FDG-PET) has assumed a valuable role in the diagnosis and longitudinal management of patients with CS, allowing for the assessment of response to treatment. Hybrid FDG-PET/CT may also be used in the evaluation of extracardiac inflammation, permitting the identification of biopsy sites for diagnostic confirmation. Herein we examine the approach to diagnosis and management of CS using multimodality imaging via a case-based review.

## Introduction

Sarcoidosis is a multiorgan system disease characterized by noncaseating granulomatous inflammation (1–3). Sarcoidosis most commonly involves the lungs or lymph nodes (2, 4). However cardiac sarcoidosis (CS) is increasingly recognized and may occur with extracardiac findings or, rarely, in isolation (4). Clinically, cardiac involvement may manifest with cardiomyopathy, arrhythmia, or atrioventricular conduction disease, or CS may remain relatively subclinical (2). While identifying CS has significant therapeutic and prognostic implications (5–7), diagnosis may be challenging, particularly in the absence of extracardiac disease.

Diagnosis of CS traditionally requires histopathologic evidence of sarcoidosis (i.e., noncaseating granulomas) either in the heart or another organ in addition to characteristic clinical and imaging findings. Several diagnostic criteria for CS have been proposed, including the Japanese Ministry of Health and Welfare (JMHW) criteria (8) and the Heart Rhythm Society (HRS) criteria (9). The widely used HRS criteria require confirmatory cardiac histopathology to make a “definite CS” diagnosis. When there is a histologic diagnosis of extracardiac sarcoidosis, a diagnosis of “probable CS” can be made with the following HRS imaging criteria: reduced left ventricular ejection fraction (LVEF) <40%, patchy uptake on dedicated ^18^F-fluorodeoxyglucose position emission tomography (FDG-PET) scan, and/or late gadolinium enhancement (LGE) on cardiac magnetic resonance imaging (CMR) (9). However, with advancements in cardiac imaging, and the limited diagnostic yield of biopsy (4, 10, 11), there has been increased reliance on imaging and clinical presentation for the diagnosis of CS (12). More recently, the revised Japanese Circulation Society updated criteria for CS to allow for a diagnosis of possible or isolated CS based on imaging characteristics, including cardiac FDG uptake, LGE on CMR, and abnormalities in ventricular wall anatomy and function (basal thinning of the interventricular septum, ventricular aneurysm, LVEF < 50%) (12). Notably, while the updated Japanese criteria still include abnormal 12-lead electrocardiogram (ECG) findings (ventricular arrhythmias, bundle branch bock, axis deviation, pathologic Q waves) as minor criteria for CS diagnosis (12), ECG has low sensitivity and specificity for CS (7). HRS guidelines include ECG as a screening tool for cardiac involvement among patients with known extracardiac sarcoidosis, where it is best used for screening in conjunction with echocardiography to increase diagnostic yield (9).

**Figure 1:**
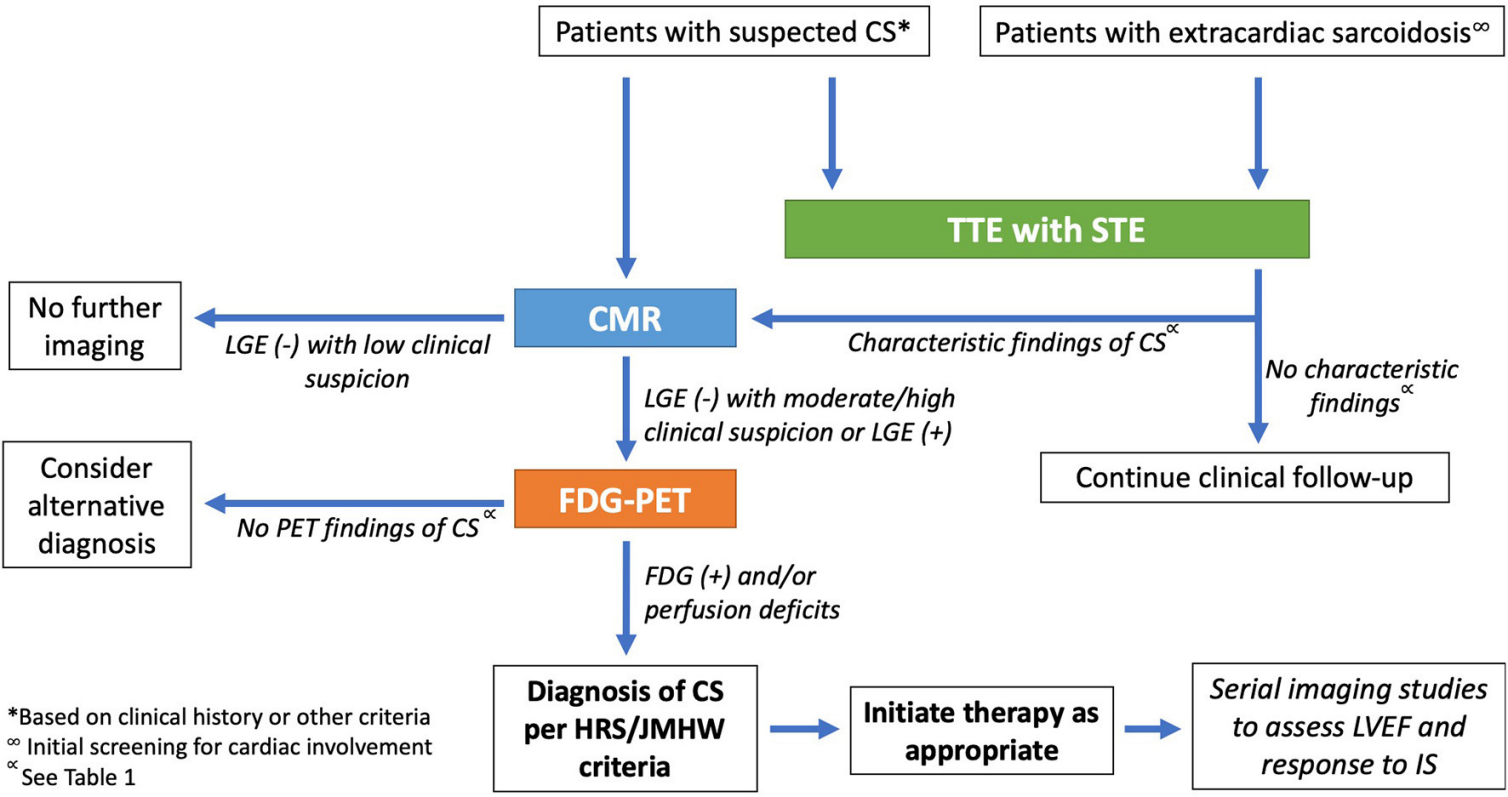
Proposed algorithm for the multimodal imaging approach to diagnosis and management of CS.

Here we provide a case-based review of multimodality cardiac imaging, specifically echocardiography, CMR, and FDG-PET, in CS, with an emphasis on diagnostic and management strategies (Figure 1). We also highlight the current limitations and challenges as well as future directions of advanced cardiac imaging in CS.

## Echocardiography

*A 42-year-old male with a history of previously treated, well controlled pulmonary sarcoidosis presents with 3 months of progressive dyspnea on exertion, weight gain and fatigue. Physical exam is notable for elevated jugular venous pressure, bilateral inspiratory rales and pitting pretibial edema. He is referred for an echocardiogram, which demonstrates low normal left ventricular systolic function with an LVEF of 50–55%, moderate concentric left ventricular (LV) hypertrophy, restrictive diastolic filling pattern (mitral inflow E/A ratio 2.2) and mild hypokinesis of the right ventricle. Global longitudinal strain (GLS) is reported at* −*6% (normal* < −*18%). Given concern for restrictive cardiomyopathy, he is referred for endomyocardial biopsy, which demonstrates fibrosis without active granulomatous inflammation. Ongoing suspicion for cardiac involvement of sarcoidosis prompts advanced cardiac imaging, ultimately confirming a diagnosis of CS. He is initiated on corticosteroids and mycophenolate mofetil*.

**Figure 2:**
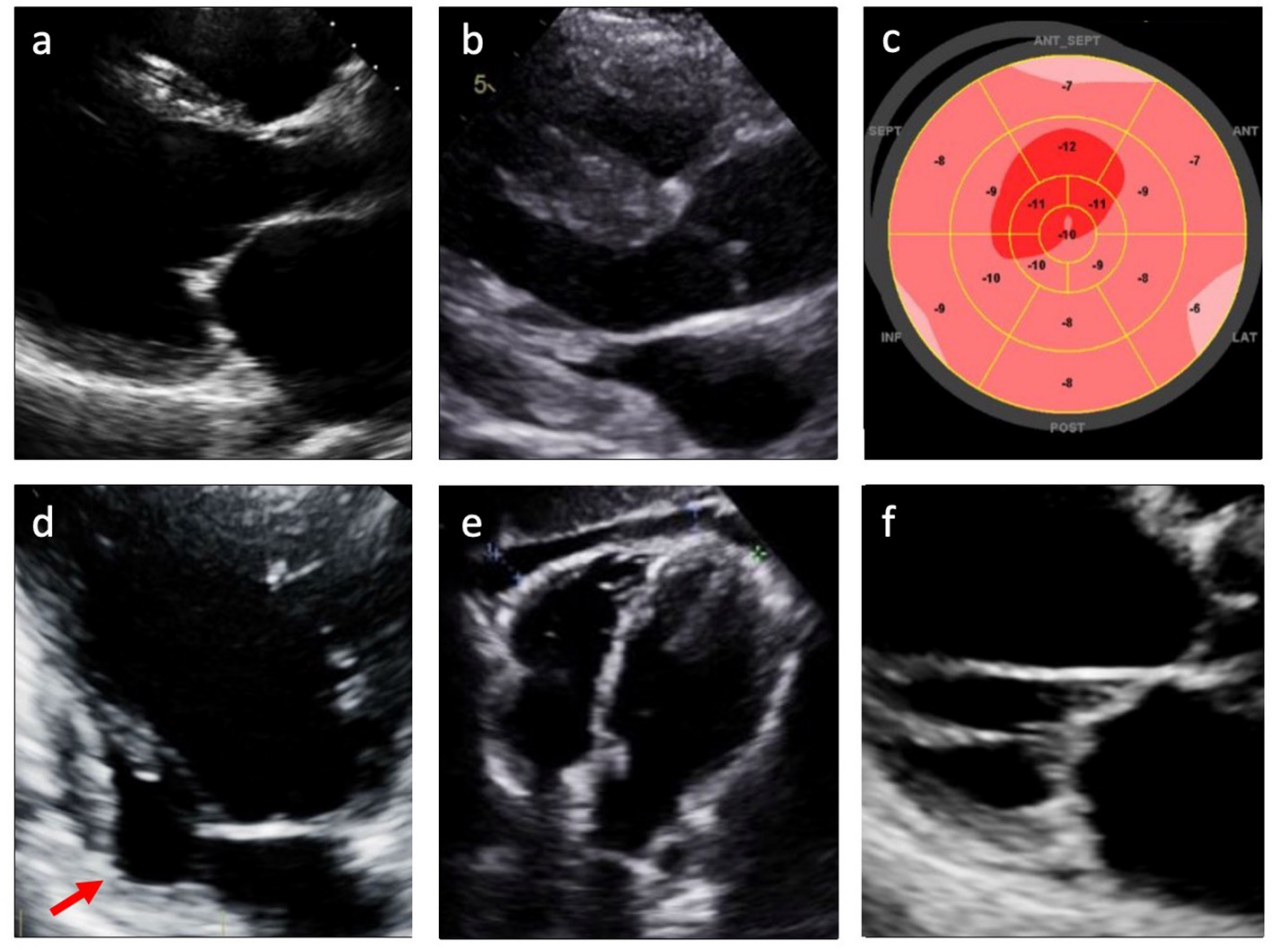
Echocardiographic findings in cardiac sarcoidosis. **(a)** Left ventricular dilatation; **(b)** Left and/or right ventricular hypertrophy; **(c)** Reduced global longitudinal strain (GLS); **(d)** Left ventricular wall aneurysm (arrow); **(e)** Pericardial effusion; **(f)** Valvular thickening or dysfunction.

Two-dimensional transthoracic echocardiography (TTE) remains a cornerstone in the investigation of patients with suspected CS (9). TTE is the only imaging modality recommended by HRS guidelines for the screening of patients with extracardiac sarcoidosis for cardiac involvement (9). Left ventricular systolic or diastolic dysfunction, ventricular dilatation, abnormal septal wall thickness, wall motion abnormalities in non-coronary distributions, ventricular aneurysms, and pericardial effusion are all findings that have been associated with CS (Figure 2) (2, 5, 7, 13, 14). Left ventricular hypertrophy and restrictive physiology may also be noted (2, 11, 15), with associated biatrial enlargement and restrictive diastolic filling pattern (as evidenced by mitral inflow pattern with *E/A* ratio ≥ 2 and findings consistent with increased left atrial pressure) (16). Several studies have identified thickening or thinning of the septal wall as a more specific finding for CS (17, 18). However, many patients with CS do not manifest any of these echocardiographic abnormalities, limiting the sensitivity of this modality for identifying CS (7, 19).

More recently, advanced techniques such as speckle tracking echocardiography (STE) have shown promise in identifying subclinical myocardial dysfunction in CS. The tracking of grayscale speckles within the myocardium over the cardiac cycle allows for assessment of myocardial deformation using measurements such as strain, or change in length compared to baseline length (20, 21). STE deformation parameters can assess mechanics at the level of the cardiomyocytes and are sensitive to histopathological changes in myocardial tissue (21). Thus, reductions in strain, globally or over a regional area of interest, can indicate underlying myocardial disease (20, 21).

CS is characterized by myocardial inflammation, fibrosis, and edema (22), pathologic changes that affect tissue function and, consequently, measures of strain before overt changes in LV function might be detected by TTE. Di Stefano and colleagues, for example, compared 23 patients with definite or probable CS and normal LV and RV systolic function with no baseline wall motion abnormalities to 97 healthy controls (23). The authors found a significant impairment in left ventricular global longitudinal strain, LVGLS (absolute LVGLS 15.9% ± 2.5 vs. 18.2% ± 2.7, *P* = 0.001) and right ventricular global longitudinal strain, RVGLS (absolute RVGLS 16.9% ± 4.5 vs. 24.1% ± 4.0, *P* = 0.0001) among those with CS (23). Notably, among the larger cohort of 83 patients with definite or probable CS in this study (including those with reduced LVEF), event rates for hospitalization or heart failure were higher in those patients with absolute LVGLS < 14% (23).

Additionally, multiple observational studies have demonstrated that reductions in GLS may be identified by STE in patients with sarcoidosis without known CS or apparent LV dysfunction, suggestive of early subclinical myocardial dysfunction (24–27). A recent meta-analysis of these studies found that LVGLS was significantly impaired in patients with extracardiac sarcoidosis and normal LV function compared with controls, and that among patients with sarcoidosis, LVGLS was significantly reduced in patients who experienced major cardiac events (28). These studies suggest a potential role for STE as a more sensitive screening tool than traditional echocardiography alone to identify patients with extracardiac sarcoidosis at increased risk for cardiac involvement.

Among patients with known CS, TTE and STE may have a role in longitudinal management. Recognition of LV dysfunction is important for implementing guideline-directed medical therapy (GDMT) for heart failure, while serial TTE may be used to monitor response to medications or potentially identify candidates for advanced heart failure therapies and devices (5, 10, 29). The role for neurohormonal blockade to prevent maladaptive LV remodeling is not well understood for patients with impaired GLS without overt LV dysfunction. The field of cardio-oncology, where preemptive use of cardioprotective medications in patients receiving cardiotoxic medications to prevent cancer treatment related cardiac dysfunction has been more extensively evaluated, may provide some insight (30, 31). For example, one study of 159 patients receiving potentially cardiotoxic chemotherapy (anthracyclines, trastuzumab, or both) showed that among patients with decreased absolute GLS by ≥ 11% relative to baseline, those who received beta blockers demonstrated improvement in GLS on follow-up (32). Additional studies are needed to explore a similar role for cardioprotective medications among patients with CS, particularly those in whom subclinical LV dysfunction is identified early on cardiac imaging.

## Cardiovascular Magnetic Resonance Imaging

*A 63-year-old African American female with a history of hypertension and dyslipidemia presents to the emergency department with 1 week of intermittent chest pressure and palpitations. ECG on arrival shows sinus rhythm with a nonspecific intraventricular conduction delay and occasional premature ventricular contractions. Serum troponin levels are undetectable. Chest x-ray is notable for an enlarged cardiac silhouette and hilar lymphadenopathy. Transthoracic echocardiogram reveals global LV systolic dysfunction with LVEF 40% and thinning of the basal septal wall. Coronary angiography shows non-obstructive coronary artery disease. She is referred for CMR, which shows midwall delayed gadolinium enhancement in the inferolateral basal septal LV, suspicious for CS. For further diagnostic evaluation, she undergoes bronchoscopy with endobronchial ultrasound-guided lymph node biopsy. Histopathology demonstrates macrophages and noncaseating granulomas. Given histologic confirmation of sarcoidosis, CMR findings and in context of borderline LV function, electrophysiology study is performed for further arrhythmic risk stratification and demonstrates inducible ventricular tachycardia. She undergoes implantable cardioverted-defibrillator (ICD) placement and initiation of immunosuppressive therapy for CS*.

**Figure 3:**
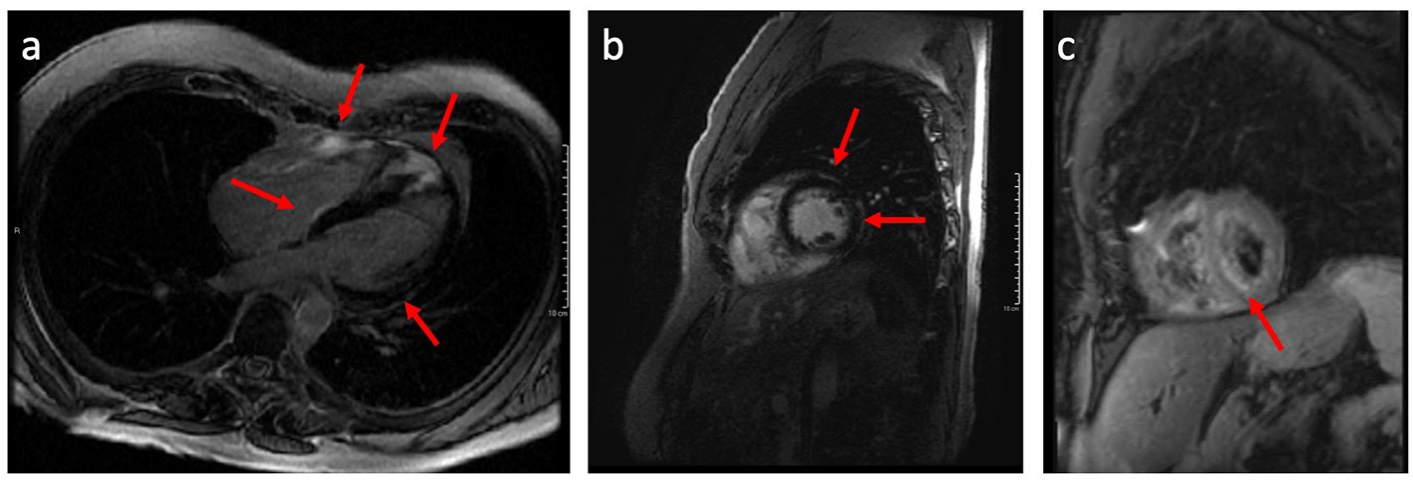
Long axis **(a)** and short axis **(b)** CMR images demonstrating late gadolinium enhancement (LGE) having a patchy non-vascular midmyocardial and sub-epicardial pattern mainly involving the basal and apical septal wall, basal-mid lateral and anterior wall, and right ventricular wall; **(c)** Black blood T-2 weighted CMR images demonstrate patchy areas of predominantly midmyocardial increased signal intensity in the left and right ventricular myocardium denoting myocardial inflammation.

CMR has established a role as a highly sensitive tool with both diagnostic and prognostic value in the management of CS. CMR has wide application in the evaluation of nonischemic cardiomyopathies, in part owing to the ability to identify myocardial fibrosis by LGE (33). Midwall and subepicardial LGE, commonly involving the basal or mid-ventricular septum, are characteristic of CS, though other patterns have been noted (Figure 3) (34–39). Lesions detected by LGE-CMR may be too small to cause conduction disturbances or LV structural or functional changes that might be identified by ECG or TTE, but nonetheless may have clinical importance (33–35). LGE-CMR has demonstrated increased sensitivity for cardiac involvement among patients with sarcoidosis when compared with JMHW criteria alone (35). In another cohort of 321 sarcoidosis patients, among whom 96 (29.9%) met HRS criteria for CS, CMR demonstrated the highest sensitivity (96.9%), specificity (100%), and area under the curve (0.984) when compared to ECG, Holter monitoring, and TTE (40). CMR can also provide comprehensive assessment of cardiac morphology and function including left and right ventricular systolic function, ventricular dimensions, wall thickness, and wall motion abnormalities (41, 42). The emerging CMR technique of strain imaging may offer another means to assess the effect of CS on LV mechanics (43). Multiple authors have investigated the use of CMR strain imaging for diagnosis and prognostication (44–46). One recent study of 76 patients with CS who underwent CMR with both LGE and longitudinal strain imaging suggested that regional longitudinal strain was not well associated with either arrhythmic phenotype (atrioventricular block vs. ventricular arrhythmia) or future adverse events compared to LGE (46); however, more data are needed to understand the potential role of CMR strain imaging in CS.

In addition to its diagnostic utility, CMR has also demonstrated prognostic power (35, 47–50). In an early study by Patel and colleagues noted above, patients with LGE on CMR had higher rates of the composite endpoint of all-cause mortality or symptomatic arrhythmia as well as higher rates of cardiac death (35). Likewise, in a larger cohort of 155 patients with systemic sarcoidosis undergoing CMR for suspected cardiac involvement, LGE was associated with an increased risk of death, aborted sudden cardiac death, or appropriate ICD firing (HR 31.6, *P* = 0.0014) on multivariate analysis (48). The presence of LGE was found to be a better independent predictor of cardiac death than LVEF, which has previously been identified as a predictor of mortality among patients with CS (48, 51, 52). A recent meta-analysis including these and similar studies, including 694 subjects in total, found an increased risk of cardiovascular death (relative risk 10.7, 95% confidence interval [CI] 1.34–86.3, *P* = 0.03) and ventricular arrhythmia (relative risk 19.5, 95% CI 2.68–143, *P* = 0.003) in LGE-positive patients compared to LGE-negative patients (49). Notably, LGE-negative patients (495/694) had low rates of cardiovascular mortality and ventricular arrhythmias, suggesting that LGE-CMR also confers a high negative predictive value and that LGE-negative patients have a favorable prognosis (49). Similarly, it has been noted that inflammation on FDG-PET in the absence of LGE on CMR identifies lower risk group for ventricular arrhythmias compared to FDG positive patients with LGE (53).

LGE-CMR has a particularly nuanced role in the decision for ICD placement among patients with CS. Persistent LVEF ≤ 35% despite optimal medical therapy and immunosuppression (if indicated), sustained ventricular tachycardia, and aborted sudden cardiac arrest remain class I indications for an ICD by the most recent HRS guidelines (9, 54), while class IIa indications include patients with LVEF ≥ 35% and syncope, evidence of myocardial scar by CMR or FDG-PET, an indication for permanent pacing, or inducible sustained ventricular arrhythmia on electrophysiological study (54). LGE-CMR may identify additional patients at increased risk of sudden cardiac death in the absence of significantly reduced LV function (9). Interestingly, several studies have identified LGE regional variations in risk of ventricular arrhythmias and sudden cardiac death (46, 55). One study of 290 patients with biopsy-proven sarcoidosis undergoing CMR for suspected cardiac involvement found that LGE in the right ventricle was independently associated with the combined endpoint of sudden cardiac death or significant ventricular arrhythmia (HR 5.43, 95% CI 1.25–23.47, *P* = 0.024) (55). Thus, CMR may prompt referral for ICD for patients with higher risk LGE features. Conversely, the 2014 HRS consensus statement indicates that absence of LGE in patients without other class I indications identifies patients who should not receive ICD therapy (class III) (9).

LGE-CMR is not without limitations. While sensitive to even small regions of fibrosis (34, 35), midwall enhancement is not specific to CS and can be seen in other nonischemic cardiomyopathies, including arrhythmogenic right ventricular cardiomyopathy (56). Though less common, transmural distribution or subepicardial and subendocardial distribution of LGE (with midwall sparing), as well as multifocal LGE may also indicate CS (5, 36). Additionally, LGE-CMR may be less sensitive in patients in earlier stages of CS, who have acute inflammation but have not yet developed myocardial fibrosis (33, 43). T2-weighted imaging may increase detection of acute inflammation, though more data are needed to understand the role of T2 mapping in CS (44, 57). CMR may be technically challenging in patients with permanent pacemakers or cardiac defibrillators (43, 58). Importantly, recent studies have demonstrated the safety of MRI in patients with non-MRI-conditional devices using safety protocols, which may mitigate this concern (59, 60). Finally, gadolinium is relatively contraindicated in patients with severe renal disease due to the risk of nephrogenic systemic fibrosis (43, 58).

## ^18^F-fluorodeoxyglucose Position Emission Tomography

*A 49-year-old male with a history of biopsy-proven pulmonary sarcoidosis and recent complete heart block status post permanent pacemaker presents in clinic for further evaluation of possible cardiac involvement of sarcoidosis. ECG demonstrates sinus rhythm with right ventricular pacing. TTE shows normal biventricular size and function. CMR reveals LGE localized to the basal septum. He is referred for cardiac FDG-PET, which demonstrates patchy FDG uptake involving the basal septal and inferolateral LV wall with co-localized perfusion defects, concerning for active CS. A course of prednisone and methotrexate are initiated and 4 months later FDG-PET scan is repeated showing near resolution of cardiac FDG uptake. Pacemaker interrogation reveals recovery of AV node conduction*.

**Figure 4:**
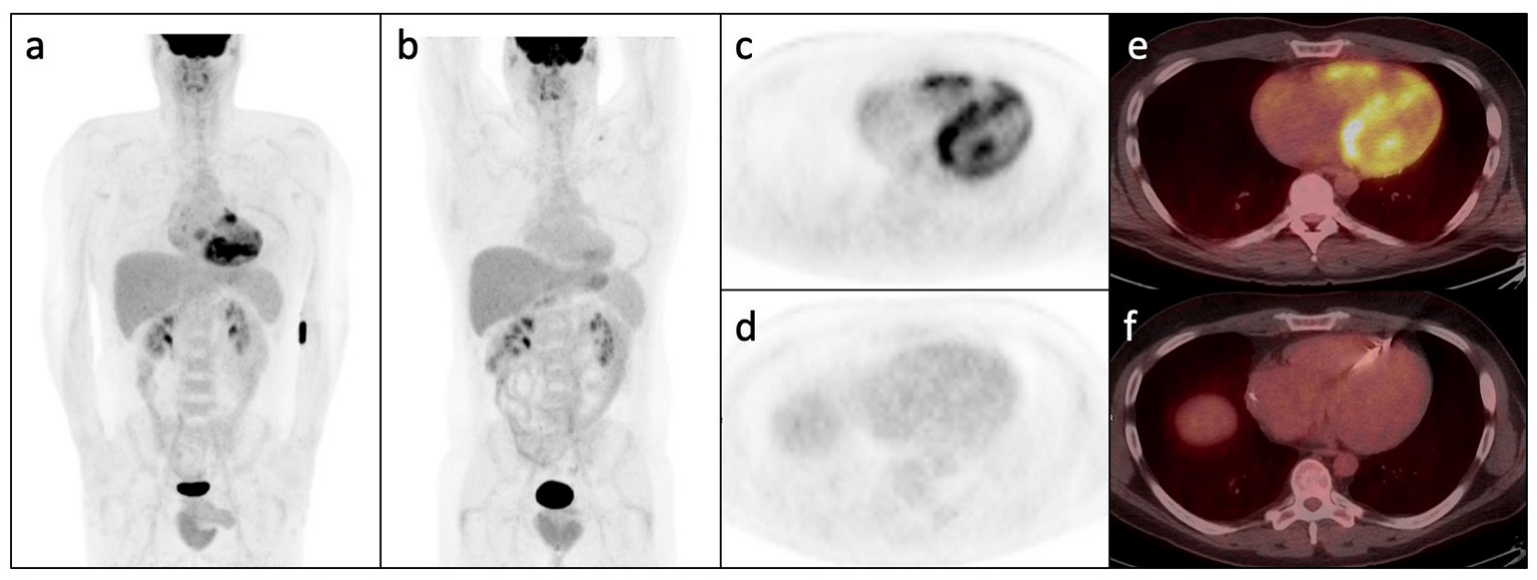
Whole-body FDG PET **(a)** and axial PET and PET/CT images **(c,e)** following appropriate pre-test preparation demonstrating abnormal patchy increase in FDG avidity within the right and left ventricular myocardium with subtle increase in right atrial FDG uptake. **(b,d,f)** Show post-treatment FDG PET/CT images in the same patient demonstrating interval resolution of the previously seen abnormal myocardial FDG uptake. Note interval ICD placement.

**Figure 5:**
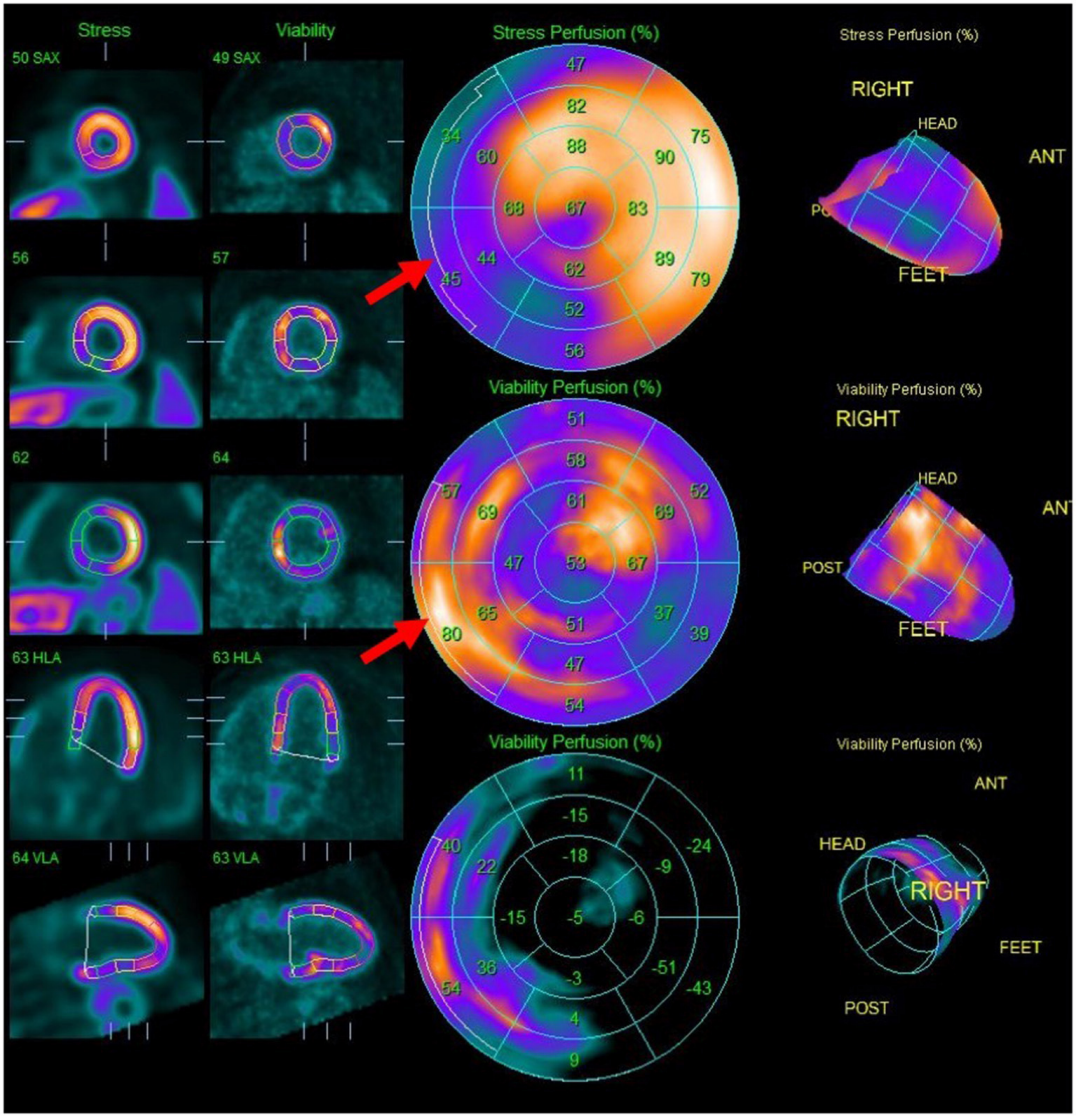
Splash images demonstrating moderate to severe transmural perfusion abnormality mainly involving the mid-base septal and inferoseptal wall (upper row), corresponding to areas of increased FDG uptake (middle row), denoting significant inflammation causing decreased perfusion (“mismatch” pattern). Note additional sites of increased FDG uptake without corresponding decreased perfusion such as in the mid-apical anterolateral wall.

FDG-PET with myocardial perfusion imaging has emerged as an important imaging modality in CS, combining assessment of active cardiac inflammation with evaluation of perfusion (Figures 4, 5) (61, 62). ^18^F-FDG is a glucose analog that is readily utilized by activated macrophages (61, 63). Accumulation of FDG by these highly metabolic inflammatory cells within active granulomas allows for visualization of active inflammation in CS (22, 64, 65). Patterns of FDG uptake associated with CS have been described as focal, focal on diffuse, or less commonly, diffuse, though diffuse FDG uptake may be difficult to interpret (9, 62). Hybrid PET/CT imaging facilitates identification of alternate sources of abnormal FDG uptake, such as malignant lesions or infections (62). Additionally, metrics to quantify FDG uptake, such as standardized uptake values (SUVs), may aid in interpretation and comparison of studies (62, 66, 67).

Importantly, whole-body PET can identify extracardiac inflammation and accessible biopsy sites to confirm histopathologic diagnosis of sarcoidosis (22, 67, 68). FDG-PET guidance can improve the diagnostic yield of noncardiac biopsy targets such as thoracic lymph nodes, which typically have higher yield than endomyocardial biopsy, especially when significantly FDG avid (67). Furthermore, assessment of the extent and activity of extracardiac involvement may have implications for treatment decisions (68).

It is recommended to combine ^18^F-FDG metabolic imaging with myocardial perfusion imaging (MPI) using rubidium-82 or N-13-ammonia (62, 67). Perfusion defects, related to changes in coronary microcirculation caused by CS, typically occur in non-coronary distributions and may represent areas of inflammation or fibrosis (62, 69). Pairing FDG and MPI patterns can provide information regarding the activity and chronicity of cardiac involvement (62, 67, 70). Active inflammation may result in FDG uptake in an area of abnormal perfusion (mismatched segment), whereas fibrosis may cause a perfusion defect in the absence of FDG uptake (11, 67, 68).

The sensitivity of FDG-PET for the diagnosis of CS has been reported as 85–100% in various studies, with a specificity ranging from 39 to 100% (62, 67). One meta-analysis of 7 studies yielded a pooled sensitivity of 89% (95% CI 79–96%) and specificity of 78% (95% CI 68–86%) (61). However, multiple authors note that estimations of specificity may be limited by the use of JMHW criteria as the standard in multiple studies, which have lower sensitivity for CS than FDG-PET (62, 67). Other diagnoses to consider in the setting of positive FDG uptake include myocardial ischemia with hibernating myocardium, other forms of myocarditis or systemic rheumatologic diseases associated with myocardial inflammation, or some arrhythmogenic cardiomyopathies (67, 71, 72).

Important for ensuring high diagnostic accuracy of FDG-PET is effective suppression of physiologic myocardial glucose uptake by shifting cardiomyocytes preferentially to fatty acid metabolism (67). Suboptimal patient preparation may lead to diffuse FDG uptake, limiting visualization of active sarcoid lesions or leading to false positive results (61, 62, 73). The most recent joint SNMMI/ASNC expert consensus statement recommendations include two high-fat (>35 g), low carbohydrate (<3 g) meals the day prior to the study followed by a 4–12 period of fasting; an 18 hour fast is an alternative option (67). The adjunctive use of unfractionated heparin immediately prior to the scan has been described (74) but was not specifically recommended in the SNMMI/ASNC document (67). A recent study investigating the use of a structured preparation protocol adhering to the new SNMMI/ASNC guidelines compared to a former less-rigorous protocol showed that a strict high-fat, low-carbohydrate diet with prolonged fasting, compliance reinforcement, and detailed instructions was highly successful in suppressing physiologic ^18^F-FDG uptake (91% among the structured protocol group vs. 78% in the standard protocol group, *P* < 0.001) (73).

Given the limitations posed by the use of ^18^F-FDG in the setting of physiologic uptake by cardiac myocytes, alternative radiotracers have been explored to improve the specificity of PET imaging in CS (75–81). One novel radiotracer of particular interest is a radiolabeled somatostatin analog (^68^Ga-somatostatin analog), which targets the somatostatin receptor (SSTR) 2 subtype that is highly expressed in sarcoid granulomas but not in normal cardiac myocytes (75). Early feasibility studies suggest somatostatin analogs may increase diagnostic accuracy compared to FDG-PET (77, 78); however, more data are needed to guide the use of this modality.

When high quality imaging can be obtained, serial FDG-PET imaging may be used to assess response to treatment and to guide management of CS. One single-center study of 32 patients with CS who underwent FDG-PET imaging before and after corticosteroid therapy demonstrated that 81% of patients had a decrease in the extent and 88% experienced a decrease in the intensity of FDG uptake on follow-up imaging (82). A separate study of 34 patients with CS who collectively underwent 128 FDG-PET scans per an institutional management protocol found that 94 (73%) of scans led to a change in therapy and 42 (33%) resulted in a decrease in prednisone dose (83). Several retrospective studies have now demonstrated the role of serial FDG-PET in guiding immunosuppression management, specifically the ability to taper corticosteroids while maintaining good cardiac disease control (83–85). While SNMMI/ASNC guidance recommends assessing change in intensity and extent of FDG uptake on follow-up studies (67), it is also worth noting that perfusion defects, which may be related to microvascular compression and local ischemia, may also resolve with treatment (62, 67). The ongoing CHASM-CS randomized clinical trial of combination prednisone/methotrexate compared to prednisone alone for initial treatment of active CS includes perfusion defects on 6-month PET scan as the primary endpoint (86). Experts recommend repeat FDG-PET imaging in a 3–6 month interval after initiation of immunosuppressive therapy to assess for improvement (which may guide tapering of corticosteroids and minimize drug related side effects) vs. stability to worsening of inflammation (possibly prompting escalation of therapy) (6, 11, 62, 68).

For patients with CS, FDG-PET imaging also conveys important prognostic information. Blankstein et al. found that among 118 patients referred for FDG-PET for evaluation of possible CS, the presence of both perfusion defects and FDG uptake was associated with increased incidence of death or sustained ventricular tachycardia (HR 3.94, 95% CI 1.50–10.31, *P* < 0.01) compared to patients with normal imaging (87). Notably, right ventricular FDG uptake was also associated with adverse events (HR 4.22, 95% CI 1.87–9.50, *P* < 0.001). Similarly, among 67 patients with CS who were referred for FDG-PET, intensity of FDG uptake (as quantified by standardized uptake values, SUV) was associated with increased incidence of cardiac events (88). Other studies have noted that decrease in inflammation on serial FDG-PET scans is associated with improvement in LVEF (89, 90). The longer-term implications however of mildly persistent FDG uptake or perfusion defects remain unknown in patients with otherwise clinically controlled CS.

## Imaging in CS: A Multimodality Approach

The pathophysiology of CS lends itself to the complementary imaging modalities of echocardiography, CMR and FDG-PET for purposes of diagnosis, management, and prognostication. A proposed algorithm for imaging in CS is provided in Figure 1. Echocardiography is highly accessible and allows an initial, urgent assessment of ventricular function, valvular disease or pericardial effusion that may point toward specific immediate management approaches. Advanced cardiac imaging allows for more nuanced CS assessment. Focal inflammation identified and quantified by FDG uptake may be prominent in early stages of the disease, whereas fibrosis occurring later in the disease course may be better assessed by superior spatial resolution of CMR. Several studies have evaluated the utility of sequential (38, 91, 92) or hybrid (93–95) CMR/PET imaging for diagnosis of CS. In the largest of these studies, 107 patients underwent both CMR and FDG-PET for evaluation of known or suspected CS and imaging findings were integrated to determine the combined likelihood of CS (no CS, possible CS, probable CS, or highly probable CS) (38). When FDG-PET results were added to findings from CMR, 48 patients (45%) were reclassified as having a higher or lower probability of CS compared to results from a single imaging study (38). Similarly, a small study of patients undergoing hybrid CMR/PET imaging resulted in high quality ^18^F-FDG and CMR images, demonstrating the value of this modality for diagnosis, prognosis, and potentially cost-saving (95). Notably, both FDG-PET and CMR are included among HRS criteria for diagnosis of CS and carry a class IIa recommendation for performing in patients with at least one abnormality detected on initial cardiac screening (history, ECG, and TTE) (9). However, given the high negative predictive value, CMR might serve as the best initial testing option—in many patients, a normal CMR might be sufficient to obviate the need for further testing (62, 68). By JMHW criteria, a clinical diagnosis of CS might be made with abnormalities on TTE and CMR in the presence of one major clinical criterion (advanced AV block, thinning of the basal interventricular septum, positive cardiac Gallium-67 uptake, or LVEF < 50%) (8). Interstitial fibrosis or monocyte infiltration on endomyocardial biopsy may also comprise a minor criterion, with identification of noncaseating granulomas confirming a histological diagnosis; however, the yield of endomyocardial biopsy is often limited (9). FDG-PET is excluded from these guidelines, with potential implications for the sensitivity of JMHW criteria for diagnosing CS (62, 67). Importantly, advanced imaging modalities of CMR and FDG-PET are both incorporated into the more recent Japanese Circulation Society guidelines as major criteria for a diagnosis of CS (12), reflecting the value of these tests in evaluating patients with suspected CS.

Beyond confirming a diagnosis, the management of CS also relies heavily on multimodality imaging. As previously detailed, FDG-PET has shown to be an effective tool for monitoring response to and tailoring immunosuppression. Serial echocardiographic evaluation is invaluable for longitudinal assessment of LV function to guide GDMT and, if needed, identify candidates for advanced therapies including left ventricular assist devices and orthotopic heart transplant (4, 5, 11). Another important decision point pertains to ICD therapy and is again highly reliant on imaging findings to guide management. Echocardiography and CMR are essential for risk stratification of patients with CS to classify those at highest risk of sudden cardiac death (4, 9, 54).

## Conclusions

CS is a disease of complex pathophysiology that is well-suited to a multimodality imaging approach for purposes of diagnosis, treatment, and prognostication. Together, TTE, CMR and FDG-PET provide complementary clinical information that allows for a comprehensive understanding of the extent of cardiac involvement for each individual patient (Table 1). Ongoing studies involving more advanced imaging techniques—including speckle-tracking echocardiography and hybrid CMR/PET imaging—may provide additional insights. Further studies are needed to best employ these more advanced modalities for optimal management of CS.

**Table 1:** Imaging modalities for the diagnosis and management of cardiac sarcoidosis.

**Imaging modality**	**Techniques**	**Findings**	**Clinical role**	**Limitations**
TTE	2D TTE STE	• Left or right ventricular systolic/diastolic dysfunction • Ventricular dilatation • Abnormal septal wall thickness • LVH • Wall motion abnormalities • Ventricular aneurysm • Pericardial effusion • Valvular dysfunction • Reduced GLS	• Initial screening of patients with ECS • Serial monitoring of LV function (for purpose of GDMT, ICD, AHF therapy) • Reduced GLS associated with adverse cardiac events	• Limited sensitivity/specificity
CMR	LGE T1/T2-mapping	• Midwall/ subepicardial LGE • Patchy, non-coronary distribution • Basal septum most commonly involved	• CS diagnosis (subacute/chronic) • Evaluation of LV morphology/function • Risk stratification (LGE associated with VA/SCD)	• May be less specific for CS • Limited sensitivity in early disease • Challenging in patients with devices • Gadolinium contraindicated in advanced CKD
FDG-PET	^18^F-FDG MPI Hybrid PET/CT Whole body PET	• Focal or focal-on-diffuse FDG uptake • FDG-avid extracardiac lesions • Perfusion defects • FDG/perfusion mismatch	• CS diagnosis (acute/chronic) • Serial imaging to assess response to/titrate of IS • Assess ECS activity • Identify non-cardiac biopsy sites • Risk stratification (FDG uptake associated with death/VA)	• Patient preparation required for adequate glucose suppression • May be less specific for CS

*AHF, advanced heart failure; CKD, chronic kidney disease; CMR, cardiac magnetic resonance imaging; ECS, extracardiac sarcoidosis; GDMT, guideline-directed medical therapy; GLS, global longitudinal strain; ICD, implantable cardioverter-defibrillator; IS, immunosuppression; LGE, late gadolinium enhancement; LVH, left ventricular hypertrophy; MPI, myocardial perfusion imaging; FDG-PET, ^18^F-fluorodeoxyglucose position emission tomography; SCD, sudden cardiac death; STE, speckle-tracking echocardiography; TTE, transthoracic echocardiogram; VA, ventricular arrhythmia*.

## Author Contributions

NG and AW conceived the design of the paper. AW completed the initial manuscript draft for review. ES assisted with design of figures and made critical revisions to the paper. NG, JC, MM, and AH made critical revisions to the intellectual content of the paper. All authors approved the final manuscript for publication.

## Conflict of Interest

The authors declare that the research was conducted in the absence of any commercial or financial relationships that could be construed as a potential conflict of interest.

## Publisher's Note

All claims expressed in this article are solely those of the authors and do not necessarily represent those of their affiliated organizations, or those of the publisher, the editors and the reviewers. Any product that may be evaluated in this article, or claim that may be made by its manufacturer, is not guaranteed or endorsed by the publisher.

## References

[1] Iannuzzi MC Rybicki BA Teirstein AS. Sarcoidosis. N Engl J Med. (2007) 357:2153–65. 10.1056/NEJMra071714 18032765

[2] Aggarwal NR Snipelisky D Young PM Gersh BJ Cooper LT Chareonthaitawee P. Advances in imaging for diagnosis and management of cardiac sarcoidosis. Eur Heart J Cardiovasc Imaging. (2015) 16:949–58. 10.1093/ehjci/jev142 26104960

[3] Drent M Crouser ED Grunewald J. Challenges of Sarcoidosis and Its Management. Longo DL, ed N Engl J Med. (2021) 385:1018–32. 10.1056/NEJMra2101555 34496176

[4] Birnie DH Nery PB Ha AC Beanlands RSB. Cardiac Sarcoidosis. J Am Coll Cardiol. (2016) 68:411–21. 10.1016/j.jacc.2016.03.605 27443438

[5] Gilotra N Okada D Sharma A Chrispin J. Management of Cardiac Sarcoidosis in 2020. Arrhythm Electrophysiol Rev. (2020) 9:182–8. 10.15420/aer.2020.09 33437485 PMC7788397

[6] Trivieri MG Spagnolo P Birnie D Liu P Drake W Kovacic JC. Challenges in Cardiac and Pulmonary Sarcoidosis: JACC State-of-the-Art Review. J Am Coll Cardiol. (2020) 76:1878–901. 10.1016/j.jacc.2020.08.042 33059834 PMC7808240

[7] Kim JS Judson MA Donnino R Gold M Cooper LT Prystowsky EN. Cardiac sarcoidosis. Am Heart J. (2009) 157:9–21. 10.1016/j.ahj.2008.09.009 19081391

[8] Hiraga H Yuwai K Hiroe M. Diagnostic standard and guidelines for sarcoidosis. Jpn J Sarcoidosis Granulomatous Disord. (2007) 27:102.

[9] Birnie DH Sauer WH Bogun F Cooper JM Culver DA Duvernoy CS. HRS expert consensus statement on the diagnosis and management of arrhythmias associated with cardiac sarcoidosis. Hear Rhythm. (2014) 11:1304–23. 10.1016/j.hrthm.2014.03.043 24819193

[10] Birnie DH Kandolin R Nery PB Kupari M. Cardiac manifestations of sarcoidosis: Diagnosis and management. Eur Heart J. (2017) 38:2663–70. 10.1093/eurheartj/ehw328 27469375

[11] Gilotra NA Griffin JM Pavlovic N Houston BA Chasler J Goetz C. Sarcoidosis-Related Cardiomyopathy: Current Knowledge, Challenges, and Future Perspectives State-of-the-Art Review. J Card Fail. (2021) 00:1–19. 10.1016/j.cardfail.2021.06.016 34260889 PMC8748280

[12] Terasaki F Yoshinaga K. New Guidelines for Diagnosis of Cardiac Sarcoidosis in Japan. Ann Nucl Cardiol. (2017) 3:42–5. 10.17996/anc.17-0004225896679

[13] Burstow DJ Tajik AJ Bailey KR DeRemee RA Taliercio CP. Two-dimensional echocardiographic findings in systemic sarcoidosis. Am J Cardiol. (1989) 63:478–82. 10.1016/0002-9149(89)90323-8 2916434

[14] Skold CM Larsen FF Rasmussen E Pehrsson SK Eklund AG. Determination of cardiac involvement in sarcoidosis by magnetic resonance imaging and Doppler echocardiography. J Intern Med. (2002) 252:465–71. 10.1046/j.1365-2796.2002.01058.x 12528765

[15] Houston BA Mukherjee M. Cardiac sarcoidosis: clinical manifestations, imaging characteristics, and therapeutic approach. Clin Med Insights Cardiol. (2014) 8s1:CMC.S15713. 10.4137/CMC.S15713 25452702 PMC4240214

[16] Nagueh SF Smiseth OA Appleton CP Byrd BF Dokainish H Edvardsen T. Recommendations for the evaluation of left ventricular diastolic function by echocardiography: an update from the american society of echocardiography and the european association of cardiovascular imaging. J Am Soc Echocardiogr. (2016) 29:277–314. 10.1016/j.echo.2016.01.011 27037982

[17] Lewin RF Mor R Spitzer S Arditti A Hellman C Agmon J. Echocardiographic evaluation of patients with systemic sarcoidosis. Am Heart J. (1985) 110:116–22. 10.1016/0002-8703(85)90524-1 4013969

[18] Yazaki Y Isobe M Hiramitsu S Morimoto S Hiroe M Omichi C. Comparison of clinical features and prognosis of cardiac sarcoidosis and idiopathic dilated cardiomyopathy. Am J Cardiol. (1998) 82:537–40. 10.1016/S0002-9149(98)00377-4 9723651

[19] Mehta D Lubitz SA Frankel Z Wisnivesky JP Einstein AJ Goldman M. Cardiac involvement in patients with sarcoidosis: Diagnostic and prognostic value of outpatient testing. Chest. (2008) 133:1426–35. 10.1378/chest.07-2784 18339784

[20] Collier P Phelan D Klein AA. Test in Context: Myocardial Strain Measured by Speckle-Tracking Echocardiography. J Am Coll Cardiol. (2017) 69:1043–56. 10.1016/j.jacc.2016.12.012 28231932

[21] Moharram MA Lamberts RR Whalley G Williams MJA Coffey S. Myocardial tissue characterisation using echocardiographic deformation imaging. Cardiovasc Ultrasound. (2019) 17:1–11. 10.1186/s12947-019-0176-9 31730467 PMC6858720

[22] Orii M Imanishi T Akasaka T. Assessment of cardiac sarcoidosis with advanced imaging modalities. Biomed Res Int. (2014) 2014:897956. 10.1155/2014/897956 25250336 PMC4163361

[23] Di Stefano C Bruno G Arciniegas Calle MC Acharya GA Fussner LM Ungprasert P. Diagnostic and predictive value of speckle tracking echocardiography in cardiac sarcoidosis. BMC Cardiovasc Disord. (2020) 20:1–10. 10.1186/s12872-019-01323-0 31959111 PMC6971954

[24] Joyce E Ninaber MK Katsanos S Debonnaire P Kamperidis V Bax JJ. Subclinical left ventricular dysfunction by echocardiographic speckle-tracking strain analysis relates to outcome in sarcoidosis. Eur J Heart Fail. (2015) 17:51–62. 10.1002/ejhf.205 25431267

[25] Bayat F Fahimi A Tavana S Tabary M Khaheshi I. Subclinical involvement of the heart and its associated factors in patients with sarcoidosis with normal systolic function using 2D speckle tracking. Echocardiography. (2020) 37:41–6. 10.1111/echo.14572 31944375

[26] Kusunose K Fujiwara M Yamada H Nishio S Saijo Y Yamada N. Deterioration of biventricular strain is an early marker of cardiac involvement in confirmed sarcoidosis. Eur Heart J Cardiovasc Imaging. (2020) 21:796–804. 10.1093/ehjci/jez235 31566217

[27] Schouver ED Moceri P Doyen D Tieulie N Queyrel V Baudouy D. Early detection of cardiac involvement in sarcoidosis with 2-dimensional speckle-tracking echocardiography. Int J Cardiol. (2017) 227:711–6. 10.1016/j.ijcard.2016.10.073 27836307

[28] Barssoum K Altibi AM Rai D Kumar A Kharsa A Chowdhury M. Speckle tracking echocardiography can predict subclinical myocardial involvement in patients with sarcoidosis: A meta-analysis. Echocardiography. (2020) 37:2061–70. 10.1111/echo.14886 33058271

[29] Yancy CW Jessup M Bozkurt B Butler J Casey DE Colvin MM. 2017 ACC/AHA/HFSA Focused Update of the 2013 ACCF/AHA Guideline for the Management of Heart Failure: A Report of the American College of Cardiology/American Heart Association Task Force on Clinical Practice Guidelines and the Heart Failure Society of Amer. Circulation. (2017) 136:e137–61. 10.1161/CIR.0000000000000509 28455343

[30] Liu JE Barac A Thavendiranathan P Scherrer-Crosbie M. Strain Imaging in Cardio-Oncology. JACC CardioOncol. (2020) 2:677–89. 10.1016/j.jaccao.2020.10.011 34396282 PMC8352045

[31] Kalam K Marwick TH. Role of cardioprotective therapy for prevention of cardiotoxicity with chemotherapy: A systematic review and meta-analysis. Eur J Cancer. (2013) 49:2900–9. 10.1016/j.ejca.2013.04.030 23706982

[32] Negishi K Negishi T Haluska BA Hare JL Plana JC Marwick TH. Use of speckle strain to assess left ventricular responses to cardiotoxic chemotherapy and cardioprotection. Eur Hear J Cardiovasc Imaging. (2014) 15:324–31. 10.1093/ehjci/jet159 24057661

[33] Patel AR Kramer CM. Role of Cardiac Magnetic Resonance in the Diagnosis and Prognosis of Nonischemic Cardiomyopathy. JACC Cardiovasc Imaging. (2017) 10:1180–93. 10.1016/j.jcmg.2017.08.005 28982571 PMC5708889

[34] Smedema JP Snoep G Van Kroonenburgh MP Van Geuns RJ Dassen WR Gorgels AP. Evaluation of the accuracy of gadolinium-enhanced cardiovascular magnetic resonance in the diagnosis of cardiac sarcoidosis. J Am Coll Cardiol. (2005) 45:1683–90. 10.1016/j.jacc.2005.01.047 15893188

[35] Patel MR Cawley PJ Heitner JF Klem I Parker MA Jaroudi WA. Detection of Myocardial Damage in Patients With Sarcoidosis. Circulation. (2009) 120:1969–77. 10.1161/CIRCULATIONAHA.109.851352 19884472 PMC2778859

[36] Sano M Satoh H Suwa K Saotome M Urushida T Katoh H. Intra-cardiac distribution of late gadolinium enhancement in cardiac sarcoidosis and dilated cardiomyopathy. World J Cardiol. (2016) 8:496. 10.4330/wjc.v8.i9.496 27721933 PMC5037324

[37] Tezuka D Terashima M Kato Y Toriihara A Hirasawa K Sasaoka T. Clinical characteristics of definite or suspected isolated cardiac sarcoidosis: Application of cardiac magnetic resonance imaging and 18F-fluoro-2-deoxyglucose positron-emission tomography/computerized tomography. J Card Fail. (2015) 21:313–22. 10.1016/j.cardfail.2014.12.004 25512195

[38] Vita T Okada DR Veillet-Chowdhury M Bravo PE Mullins E Hulten E. Complementary value of cardiac magnetic resonance imaging and positron emission tomography/computed tomography in the assessment of cardiac sarcoidosis. Circ Cardiovasc Imaging. (2018) 11:e007030. 10.1161/CIRCIMAGING.117.007030 29335272 PMC6381829

[39] Nagao S Watanabe H Sobue Y Kodama M Tanaka J Tanabe N. Electrocardiographic abnormalities and risk of developing cardiac events in extracardiac sarcoidosis. Int J Cardiol. (2015) 189:1–5. 10.1016/j.ijcard.2015.03.175 25885865

[40] Kouranos V Tzelepis GE Rapti A Mavrogeni S Aggeli K Douskou M. Complementary role of CMR to conventional screening in the diagnosis and prognosis of cardiac sarcoidosis. JACC Cardiovasc Imaging. (2017) 10:1437–47. 10.1016/j.jcmg.2016.11.019 28330653

[41] Hundley WG Bluemke DA Finn JP Flamm SD Fogel MA Friedrich MG. ACCF/ACR/AHA/NASCI/SCMR 2010 expert consensus document on cardiovascular magnetic resonance. A Report of the American College of Cardiology Foundation Task Force on Expert Consensus Documents. J Am Coll Cardiol. (2010) 55:2614–62. 10.1016/j.jacc.2009.11.011 20513610 PMC3042771

[42] Hulten E Aslam S Osborne M Abbasi S Bittencourt MS Blankstein R. Cardiac sarcoidosis-state of the art review. Cardiovasc Diagn Ther. (2016) 6:50–63. 10.3978/j.issn.2223-3652.2015.12.13 26885492 PMC4731586

[43] Tadic M Cuspidi C Saeed S Milojevic B Milojevic IG. The role of cardiac magnetic resonance in diagnosis of cardiac sarcoidosis. Heart Fail Rev. (2021) 26:653–60. 10.1007/s10741-020-10035-z 33025413

[44] Puntmann VO Isted A Hinojar R Foote L Carr-White G Nagel E. T1 and T2 Mapping in recognition of early cardiac involvement in systemic sarcoidosis. Radiology. (2017) 285:63–72. 10.1148/radiol.2017162732 28448233

[45] Dabir D Meyer D Kuetting D Luetkens J Homsi R Pizarro C. Diagnostic value of cardiac magnetic resonance strain analysis for detection of cardiac sarcoidosis. RoFo Fortschritte auf dem Gebiet der Rontgenstrahlen und der Bildgeb Verfahren. (2018) 190:712–21. 10.1055/a-0598-5099 30045396

[46] Okada DR Xie E Assis F Smith J Derakhshan A Gowani Z. Regional abnormalities on cardiac magnetic resonance imaging and arrhythmic events in patients with cardiac sarcoidosis. J Cardiovasc Electrophysiol. (2019) 30:1967–76. 10.1111/jce.14082 31328324

[47] Nadel J Lancefield T Voskoboinik A Taylor AJ. Late gadolinium enhancement identified with cardiacmagnetic resonance imaging in sarcoidosis patients is associated with long-term ventricular arrhythmia and sudden cardiac death. Eur Heart J Cardiovasc Imaging. (2015) 16:634–41. 10.1093/ehjci/jeu294 25617029

[48] Greulich S Deluigi CC Gloekler S Wahl A Zürn C Kramer U. CMR imaging predicts death and other adverse events in suspected cardiac sarcoidosis. JACC Cardiovasc Imaging. (2013) 6:501–11. 10.1016/j.jcmg.2012.10.021 23498675

[49] Hulten E Agarwal V Cahill M Cole G Vita T Parrish S. Presence of late gadolinium enhancement by cardiac magnetic resonance among patients with suspected cardiac sarcoidosis is associated with adverse cardiovascular prognosis. Circ Cardiovasc Imaging. (2016) 9:e005001. 10.1161/CIRCIMAGING.116.005001 27621357 PMC5449111

[50] Coleman GC Shaw PW Balfour PC Gonzalez JA Kramer CM Patel AR. Prognostic value of myocardial scarring on CMR in patients with cardiac sarcoidosis. JACC Cardiovasc Imaging. (2017) 10:411–20. 10.1016/j.jcmg.2016.05.009 27450877 PMC5237422

[51] Chiu CZ Nakatani S Zhang G Tachibana T Ohmori F Yamagishi M. Prevention of left ventricular remodeling by long-term corticosteroid therapy in patients with cardiac sarcoidosis. Am J Cardiol. (2005) 95:143–6. 10.1016/j.amjcard.2004.08.083 15619415

[52] Kandolin R Lehtonen J Airaksinen J Vihinen T Miettinen H Ylitalo K. Cardiac Sarcoidosis. Circulation. (2015) 131:624–32. 10.1161/CIRCULATIONAHA.114.011522 25527698

[53] Gowani Z Habibi M Okada DR Smith J Derakhshan A Zimmerman SL. Utility of cardiac magnetic resonance imaging versus cardiac positron emission tomography for risk stratification for ventricular arrhythmias in patients with cardiac sarcoidosis. Am J Cardiol. (2020) 134:123–9. 10.1016/j.amjcard.2020.08.007 32950203

[54] Al-Khatib SM Stevenson WG Ackerman MJ Bryant WJ Callans DJ Curtis AB. 2017 AHA/ACC/HRS Guideline for management of patients with ventricular arrhythmias and the prevention of sudden cardiac death: a Report of the American College of Cardiology/American Heart Association Task Force on Clinical Practice Guidelines and the Heart Rhythm Society. Heart Rhythm. (2018) 15:e73–189. 10.1016/j.hrthm.2017.10.036 29097319

[55] Velangi PS Chen KHA Kazmirczak F Okasha O Von Wald L Roukoz H. Right ventricular abnormalities on cardiovascular magnetic resonance imaging in patients with sarcoidosis. JACC Cardiovasc Imaging. (2020) 13:1395–405. 10.1016/j.jcmg.2019.12.011 31954639 PMC9303493

[56] Cipriani A Bauce B De Lazzari M Rigato I Bariani R Meneghin S. Arrhythmogenic right ventricular cardiomyopathy: Characterization of left ventricular phenotype and differential diagnosis with dilated cardiomyopathy. J Am Heart Assoc. (2020) 9:1–16. 10.1161/JAHA.119.014628 32114891 PMC7335583

[57] Crouser ED Ono C Tran T He X Raman SV. Improved detection of cardiac sarcoidosis using magnetic resonance with myocardial T2 mapping. Am J Respir Crit Care Med. (2014) 189:109–12. 10.1164/rccm.201309-1668LE 24381994 PMC3919128

[58] Zhang J Li Y Xu Q Xu B Wang H. Cardiac magnetic resonance imaging for diagnosis of cardiac sarcoidosis: a meta-analysis. Can Respir J. (2018) 2018:7457369. 10.1155/2018/7457369 30651895 PMC6311842

[59] Nazarian S Hansford R Rahsepar AA Weltin V McVeigh D Gucuk Ipek E. Safety of magnetic resonance imaging in patients with cardiac devices. N Engl J Med. (2017) 377:2555–64. 10.1056/NEJMoa1604267 29281579 PMC5894885

[60] Russo RJ Costa HS Silva PD Anderson JL Arshad A Biederman RW. Assessing the risks associated with mri in patients with a pacemaker or defibrillator. N Engl J Med. (2017) 376:755–64. 10.1056/NEJMoa1603265 28225684

[61] Youssef G Leung E Mylonas I Nery P Williams K Wisenberg G. The use of 18F-FDG PET in the diagnosis of cardiac sarcoidosis: a systematic review and metaanalysis including the Ontario experience. J Nucl Med. (2012) 53:241–8. 10.2967/jnumed.111.090662 22228794

[62] Slart RHJA Glaudemans AWJM Lancellotti P Hyafil F Blankstein R Schwartz RG. A joint procedural position statement on imaging in cardiac sarcoidosis: from the Cardiovascular and Inflammation & Infection Committees of the European Association of Nuclear Medicine, the European Association of Cardiovascular Imaging, and the American. J Nucl Cardiol. (2018) 25:298–319. 10.1007/s12350-017-1043-4 29043557

[63] Koiwa H Tsujino I Ohira H Yoshinaga K Otsuka N Nishimura M. Imaging of cardiac sarcoid lesions using fasting cardiac 18F-fluorodeoxyglucose positron emission tomography: An autopsy case. Circulation. (2010) 122:535–6. 10.1161/CIRCULATIONAHA.110.952184 20679583

[64] Brudin LH Valind S-O Rhodes CG Pantin CF Sweatman M Jones T. Fluorine-18 deoxyglucose uptake in sarcoidosis measured with positron emission tomography. Eur J Nucl Med. (1994) 21:297–305. 10.1007/BF00947964 8005153

[65] Lewis PJ Salama A. Uptake of fluorine-18-fluorodeoxyglucose in sarcoidosis. J Nucl Med. (1994) 35:1647–9. http://www.ncbi.nlm.nih.gov/pubmed/7931664 7931664

[66] Ahmadian A Brogan A Berman J Sverdlov AL Mercier G Mazzini M. Quantitative interpretation of FDG PET/CT with myocardial perfusion imaging increases diagnostic information in the evaluation of cardiac sarcoidosis. J Nucl Cardiol. (2014) 21:925–39. 10.1007/s12350-014-9901-9 24879453

[67] Chareonthaitawee P Beanlands RS Chen W Dorbala S Miller EJ Murthy VL. Joint SNMMI-ASNC expert consensus document on the role of 18F-FDG PET/CT in carDiac sarcoid detection and therapy monitoring writing group. J Nucl Med. (2017) 58:1341–53. 10.2967/jnumed.117.196287 28765228 PMC6944184

[68] Blankstein R Waller AH. Evaluation of known or suspected cardiac sarcoidosis. Circ Cardiovasc Imaging. (2016) 9:e000867. 10.1161/CIRCIMAGING.113.000867 26926267

[69] Keijsers RGM Grutters JC. In which patients with sarcoidosis is FDG PET/CT Indicated? J Clin Med. (2020) 9:890. 10.3390/jcm9030890 32213991 PMC7141490

[70] Okumura W Iwasaki T Toyama T Iso T Arai M Oriuchi N. Usefulness of fasting 18F-FDG PET in identification of cardiac sarcoidosis. J Nucl Med. (2004) 45:1989–98. Available online at: http://jnm.snmjournals.org/cgi/reprint/45/12/1989%0Ahttp://ovidsp.ovid.com/ovidweb.cgi?T=JS&PAGE=reference&D=emed8&NEWS=N&AN=47611626 15585472

[71] Protonotarios A Wicks E Ashworth M Stephenson E Guttmann O Savvatis K. Prevalence of 18F-fluorodeoxyglucose positron emission tomography abnormalities in patients with arrhythmogenic right ventricular cardiomyopathy. Int J Cardiol. (2019) 284:99–104. 10.1016/j.ijcard.2018.10.083 30409737

[72] Tung R Bauer B Schelbert H Lynch JP Auerbach M Gupta P. Incidence of abnormal positron emission tomography in patients with unexplained cardiomyopathy and ventricular arrhythmias: the potential role of occult inflammation in arrhythmogenesis. Hear Rhythm. (2015) 12:2488–98. 10.1016/j.hrthm.2015.08.014 26272522 PMC4656080

[73] Christopoulos G Jouni H Acharya GA Blauwet LA Kapa S Bois J. Suppressing physiologic 18-fluorodeoxyglucose uptake in patients undergoing positron emission tomography for cardiac sarcoidosis: the effect of a structured patient preparation protocol. J Nucl Cardiol. (2021) 28:661–71. 10.1007/s12350-019-01746-4 31111450

[74] Masuda A Naya M Manabe O Magota K Yoshinaga K Tsutsui H. Administration of unfractionated heparin with prolonged fasting could reduce physiological 18F-fluorodeoxyglucose uptake in the heart. Acta radiol. (2016) 57:661–8. 10.1177/0284185115600916 26339041

[75] Saric P Young K Rodriguez-Porcel M Chareonthaitawee P PET. Imaging in cardiac sarcoidosis: a narrative review with focus on novel PET tracers. Pharmaceuticals. (2021) 14:1286. 10.3390/ph14121286 34959686 PMC8704408

[76] Kircher M Lapa C. Novel noninvasive nuclear medicine imaging techniques for cardiac inflammation. Curr Cardiovasc Imaging Rep. (2017) 10:6. 10.1007/s12410-017-9400-x 28357026 PMC5352761

[77] Lapa C Reiter T Kircher M Schirbel A Werner RA Pelzer T. Somatostatin receptor based PET/CT in patients with the suspicion of cardiac sarcoidosis: an initial comparison to cardiac MRI. Oncotarget. 7:77807–14. 10.18632/oncotarget.12799 27780922 PMC5363622

[78] Gormsen LC Haraldsen A Kramer S Dias AH Kim WY Borghammer P. A dual tracer 68Ga-DOTANOC PET/CT and 18F-FDG PET/CT pilot study for detection of cardiac sarcoidosis. EJNMMI Res. (2016) 6:52. 10.1186/s13550-016-0207-6 27316444 PMC4912521

[79] Reiter T Werner RA Bauer WR Lapa C. Detection of cardiac sarcoidosis by macrophage-directed somatostatin receptor 2-based positron emission tomography/computed tomography. Eur Heart J. (2015) 36:2404. 10.1093/eurheartj/ehv278 26093640

[80] Manabe O Hirata K Shozo O Shiga T Uchiyama Y Kobayashi K. 18F-fluoromisonidazole (FMISO) PET may have the potential to detect cardiac sarcoidosis. J Nucl Cardiol. (2017) 24:329–31. 10.1007/s12350-016-0495-2 27071998

[81] Norikane T Yamamoto Y Maeda Y Noma T Nishiyama Y. 18F-FLT PET imaging in a patient with sarcoidosis with cardiac involvement. Clin Nucl Med. (2015) 40:433–4. 10.1097/RLU.0000000000000653 25546198

[82] Okada DR Saad E Wand AL Griffin JM Kasper EK Chen EH. Effect of Corticosteroid Dose and Duration on 18-Fluorodeoxyglucose Positron Emission Tomography in Cardiac Sarcoidosis. JACC Cardiovasc Imaging. (2020) 13:1280–2. 10.1016/j.jcmg.2019.12.013 31954655

[83] Ning N Guo HH Iagaru A Mittra E Fowler M Witteles R. Serial cardiac FDG-PET for the diagnosis and therapeutic guidance of patients with cardiac sarcoidosis. J Card Fail. (2019) 25:307–11. 10.1016/j.cardfail.2019.02.018 30825644

[84] Griffin JM Chasler J Wand AL Okada DR Smith JN Saad E. Management of cardiac sarcoidosis using mycophenolate mofetil as a steroid-sparing agent. J Card Fail. (2021) 27:1348–58. 10.1016/j.cardfail.2021.06.010 34166800

[85] Gilotra NA Wand AL Pillarisetty A Devraj M Pavlovic N Ahmed S. Clinical and imaging response to tumor necrosis factor alpha inhibitors in treatment of cardiac sarcoidosis: a multicenter experience. J Card Fail. (2021) 27:83–91. 10.1016/j.cardfail.2020.08.013 32889044 PMC8350936

[86] Birnie D Beanlands RSB Nery P Aaron SD Culver DA DeKemp RA. Cardiac Sarcoidosis multi-center randomized controlled trial (CHASM CS- RCT). Am Heart J. (2020) 220:246–52. 10.1016/j.ahj.2019.10.003 31911261 PMC7367280

[87] Blankstein R Osborne M Naya M Waller A Kim CK Murthy VL. Cardiac positron emission tomography enhances prognostic assessments of patients with suspected cardiac sarcoidosis. J Am Coll Cardiol. (2014) 63:329–36. 10.1016/j.jacc.2013.09.022 24140661 PMC3955730

[88] Flores RJ Flaherty KR Jin Z Bokhari S. The prognostic value of quantitating and localizing F-18 FDG uptake in cardiac sarcoidosis. J Nucl Cardiol. (2020) 27:2003–10. 10.1007/s12350-018-01504-y 30421379

[89] Osborne MT Hulten EA Singh A Waller AH Bittencourt MS Stewart GC. Reduction in 18F-fluorodeoxyglucose uptake on serial cardiac positron emission tomography is associated with improved left ventricular ejection fraction in patients with cardiac sarcoidosis. J Nucl Cardiol. (2014) 21:166–74. 10.1007/s12350-013-9828-6 24307261

[90] Muser D Santangeli P Castro SA Liang JJ Enriquez A Werner TJ. Prognostic role of serial quantitative evaluation of 18F-fluorodeoxyglucose uptake by PET/CT in patients with cardiac sarcoidosis presenting with ventricular tachycardia. Eur J Nucl Med Mol Imaging. (2018) 45:1394–404. 10.1007/s00259-018-4001-8 29610956

[91] Bravo PE Raghu G Rosenthal DG Elman S Petek BJ Soine LA. Risk assessment of patients with clinical manifestations of cardiac sarcoidosis with positron emission tomography and magnetic resonance imaging. Int J Cardiol. (2017) 241:457–62. 10.1016/j.ijcard.2017.03.033 28318664 PMC5469686

[92] Kebed KY Carter SV Flatley E Ward RP Moss JD Appelbaum DE. Prevalence of newly diagnosed sarcoidosis in patients with ventricular arrhythmias: a cardiac magnetic resonance and 18F-FDG cardiac PET study. Int J Cardiovasc Imaging. (2021) 37:1361–9. 10.1007/s10554-020-02090-2 33225427 PMC8035303

[93] Wicks EC Menezes LJ Barnes A Mohiddin SA Sekhri N Porter JC. Diagnostic accuracy and prognostic value of simultaneous hybrid 18 F-fluorodeoxyglucose positron emission tomography/magnetic resonance imaging in cardiac sarcoidosis. Eur Heart J Cardiovasc Imaging. (2018) 19:757–67. 10.1093/ehjci/jex340 29319785

[94] Dweck MR Abgral R Trivieri MG Robson PM Karakatsanis N Mani V. Hybrid magnetic resonance imaging and positron emission tomography with fluorodeoxyglucose to diagnose active cardiac sarcoidosis. JACC Cardiovasc Imaging. (2018) 11:94–107. 10.1016/j.jcmg.2017.02.021 28624396 PMC5995315

[95] Wisenberg G Thiessen JD Pavlovsky W Butler J Wilk B Prato FS. Same day comparison of PET/CT and PET/MR in patients with cardiac sarcoidosis. J Nucl Cardiol. (2020) 27:2118–29. 10.1007/s12350-018-01578-8 30603887 PMC7749056

